# Supplementary material for: Maternal body mass index in early pregnancy is associated with overweight and obesity in children up to 16 years of age
Source: PLoS One. 2022 Oct 6;17(10):e0275542. doi: 10.1371/journal.pone.0275542 (PMC9536626; doi:10.1371/journal.pone.0275542)
Supplement: S1 Table — (DOCX) [file pone.0275542.s001.docx]

**Table S1.** Relative-risk ratios (RRR) for 4, 7, 10, 13, and 16-year old children to be classified as underweight, overweight or obese as compared to normal weight, per 5 unit increase in maternal body mass index.

|  | Total |  | Boys |  | Girls |  |
| --- | --- | --- | --- | --- | --- | --- |
|  | RRR (95% CI) | p-value | RRR (95% CI) | p-value | RRR (95% CI) | p-value |
| 4 years old |  |  |  |  |  |  |
| Unadjusted model |  |  |  |  |  |  |
| Normal weight | 1 (reference) |  | 1 (reference) |  | 1 (reference) |  |
| Underweight | 0.84 (0.42–1.66) | 0.61 | 0.77 (0.30–2.01) | 0.60 | 0.97 (0.33–2.90) | 0.96 |
| Overweight | 2.23 (1.57–3.19) | <0.001 | 2.89 (1.81–4.63) | <0.001 | 2.05 (1.13–3.74) | 0.018 |
| Obesity | 4.25 (1.87–9.64) | 0.001 | 8.06 (1.45–44.91) | 0.017 | 6.07 (1.03–35.71) | 0.046 |
| Adjusted model |  |  |  |  |  |  |
| Normal weight | 1 (reference) |  | 1 (reference) |  | 1 (reference) |  |
| Underweight | 0.84 (0.42–1.68)) | 0.63 | 0.80 (0.31–2.10) | 0.66 | 0.90 (0.29–2.84) | 0.86 |
| Overweight | 2.22 (1.55–3.19) | <0.001 | 2.91 (1.71–4.95) | <0.001 | 2.24 (1.16–4.30) | 0.016 |
| Obesity | 3.96 (1.97–7.98) | <0.001 | 4.61 (1.59–13.32) | 0.005 | 7.12 (0.70–72.53) | 0.097 |
| 7 years old |  |  |  |  |  |  |
| Unadjusted model |  |  |  |  |  |  |
| Normal weight | 1 (reference) |  | 1 (reference) |  | 1 (reference) |  |
| Underweight | 0.77 (0.37–1.62) | 0.49 | 0.46 (0.18–1.20) | 0.11 | 1.83 (0.52–6.48) | 0.35 |
| Overweight | 2.39 (1.61–3.54) | <0.001 | 2.51 (1.56–4.04) | <0.001 | 2.25 (1.08–4.71) | 0.031 |
| Obesity | 5.61 (3.41–9.25) | <0.001 | 5.54 (2.93–10.45) | <0.001 | 7.44 (3.14–17.61) | <0.001 |
| Adjusted model |  |  |  |  |  |  |
| Normal weight | 1 (reference) |  | 1 (reference) |  | 1 (reference) |  |
| Underweight | 0.80 (0.39–1.63) | 0.54 | 0.49 (0.21–1.14) | 0.097 | 1.51 (0.59–3.86) | 0.39 |
| Overweight | 2.34 (1.55–3.53) | <0.001 | 2.43 (1.47–4.03) | 0.001 | 2.10 (0.96–4.59) | 0.063 |
| Obesity | 5.39 (3.24–8.97) | <0.001 | 5.34 (2.90–9.83) | <0.001 | 9.41 (3.22–27.52) | <0.001 |
| 10 years old |  |  |  |  |  |  |
| Unadjusted model |  |  |  |  |  |  |
| Normal weight | 1 (reference) |  | 1 (reference) |  | 1 (reference) |  |
| Underweight | 0.53 (0.27–1.05) | 0.068 | 0.49 (0.20–1.23) | 0.13 | 0.57 (0.21–1.59) | 0.29 |
| Overweight | 2.62 (1.76–3.89) | <0.001 | 2.94 (1.77–4.88) | <0.001 | 2.40 (1.21–4.78) | 0.012 |
| Obesity | 8.74 (3.34–22.90) | <0.001 | 16.13 (3.97–65.60) | <0.001 | 2.65 (0.95–7.40) | 0.063 |
| Adjusted model |  |  |  |  |  |  |
| Normal weight | 1 (reference) |  | 1 (reference) |  | 1 (reference) |  |
| Underweight | 0.51 (0.26–1.02) | 0.056 | 0.46 (0.19–1.13) | 0.089 | 0.45 (0.15–1.28) | 0.13 |
| Overweight | 2.68 (1.77–4.07) | <0.001 | 2.88 (1.70–4.88) | <0.001 | 2.72 (1.29–5.73) | 0.008 |
| Obesity | 8.13 (3.08–21.48) | <0.001 | 12.07 (3.08–47.26) | <0.001 | 2.20 (1.04–4.66) | 0.040 |
| 13 years old |  |  |  |  |  |  |
| Unadjusted model |  |  |  |  |  |  |
| Normal weight | 1 (reference) |  | 1 (reference) |  | 1 (reference) |  |
| Underweight | 0.44 (0.18–1.04) | 0.063 | 0.45 (0.11–1.89) | 0.28 | 0.44 (0.14–1.34) | 0.15 |
| Overweight | 2.43 (1.65–3.59) | <0.001 | 2.06 (1.31–3.22) | 0.002 | 3.50 (1.67–7.36) | 0.001 |
| Obesity | 8.64 (3.78–19.71) | <0.001 | 7.98 (3.35–18.98) | <0.001 | 6.98 (0.73–66.52) | 0.091 |
| Adjusted model |  |  |  |  |  |  |
| Normal weight | 1 (reference) |  | 1 (reference) |  | 1 (reference) |  |
| Underweight | 0.42 (0.19–0.93) | 0.033 | 0.37 (0.10–1.33) | 0.13 | 0.43 (0.14–1.30) | 0.13 |
| Overweight | 2.38 (1.58–3.57) | <0.001 | 2.09 (1.29–3.38) | 0.003 | 3.75 (1.62–8.69) | 0.002 |
| Obesity | 8.76 (3.67–20.93) | <0.001 | 7.58 (3.28–17.51) | <0.001 | 9.18 (0.79–107.26) | 0.077 |
| 16 years old |  |  |  |  |  |  |
| Unadjusted model |  |  |  |  |  |  |
| Normal weight | 1 (reference) |  | 1 (reference) |  | 1 (reference) |  |
| Underweight | 0.57 (0.24–1.32) | 0.19 | 0.58 (0.18–1.91) | 0.37 | 0.54 (0.16–1.81) | 0.32 |
| Overweight | 2.59 (1.68–4.01) | <0.001 | 2.71 (1.56–4.71) | <0.001 | 2.02 (0.89–4.62) | 0.094 |
| Obesity | 5.81 (3.19–10.57) | <0.001 | 6.55 (3.33–12.89) | <0.001 | 3.18 (0.65–15.58) | 0.15 |
| Adjusted model |  |  |  |  |  |  |
| Normal weight | 1 (reference) |  | 1 (reference) |  | 1 (reference) |  |
| Underweight | 0.69 (0.32–1.49) | 0.34 | 0.76 (0.28–2.07) | 0.59 | 0.64 (0.21–1.93) | 0.43 |
| Overweight | 2.88 (1.84–4.52) | <0.001 | 3.05 (1.69–5.51) | <0.001 | 2.20 (0.95–5.09) | 0.065 |
| Obesity | 6.66 (3.58–12.37) | <0.001 | 8.14 (3.95–16.76) | <0.001 | 5.49 (0.88–34.25) | 0.068 |

The adjusted model was adjusted for maternal age, smoking status, and parity.
